# Recent Progress on Immunotherapy for Breast Cancer: Tumor Microenvironment, Nanotechnology and More

**DOI:** 10.3389/fbioe.2021.680315

**Published:** 2021-06-02

**Authors:** Yang Li, Wenfang Miao, Doudou He, Siqi Wang, Jianjuan Lou, Yanni Jiang, Shouju Wang

**Affiliations:** Department of Radiology, The First Affiliated Hospital of Nanjing Medical University, Nanjing, China

**Keywords:** breast cancer, tumor microenvironment, nanotechnology, immune checkpoint inhibitors, cancer vaccine, adoptive cell therapy, immunotherapy

## Abstract

Immunotherapy is a *major emerging* treatment for breast cancer (BC). However, not all breast cancer patients derive benefit from immunotherapy. Predictive biomarkers of immunotherapy, such as tumor mutation burden and tumor-infiltrating lymphocytes, are promising to stratify the patients with BC and optimize the therapeutic effect. Various targets of the immune response pathway have also been explored to expand the modalities of immunotherapy. The use of nanotechnology for the imaging of predictive biomarkers and the combination with other therapeutic modalities presents a number of advantages for the immunotherapy of BC. In this review, we summary the emerging therapeutic modalities of immunotherapy, present prominent examples of immunotherapy in BC, and discuss the future opportunity of nanotechnology in the immunotherapy of BC.

## Introduction

Breast cancer (BC) is one of the most common malignancies in women, which was considered a non-immunogenic tumor with a low presentation of immunogenic peptides and, therefore, not suitable for immunotherapies (Li et al., 2018). A growing number of studies have found that patients selected by biomarkers like the expression of programmed death protein 1 ligand (PD-L1) demonstrate increased benefit in immunotherapy, particularly in combination therapies, prompting intensive research on combination therapy and such biomarkers in the context of breast cancer.

Triple-negative breast cancer (TNBC), with low expression of hormone receptor (HR) and human epidermal growth factor receptor 2 (HER2) is described with high immunogenicity among BC subtypes. As the report of the phase III Impassion 130 trial published, renewed attention and research directions have been attracted toward the immunotherapy for breast cancer, especially on TNBC (Bayraktar et al., 2019). Furthermore, accumulated progress in the knowledge of molecular mechanisms of the interaction between tumor and immune system have allowed immunotherapy to serve either as a new therapeutic option or as an adjunct to existing treatments (surgery, radiotherapy, chemotherapy, and targeted therapy) to improve the therapeutic effectiveness of BC.

Frustratingly, lack of real-time imaging guidance, low tumor concentration, and complex tumor heterogeneity dampen the outcome of immunotherapy. Nanotechnology, for its unique physical, chemical properties and ability to deliver a variety of drugs, could provide molecular imaging guidance (Zhong et al., 2019) for BC immunotherapy and reprogram the TME of BC (Yang et al., 2020), and thus may become the breaking point of immunotherapy.

**FIGURE 1:**
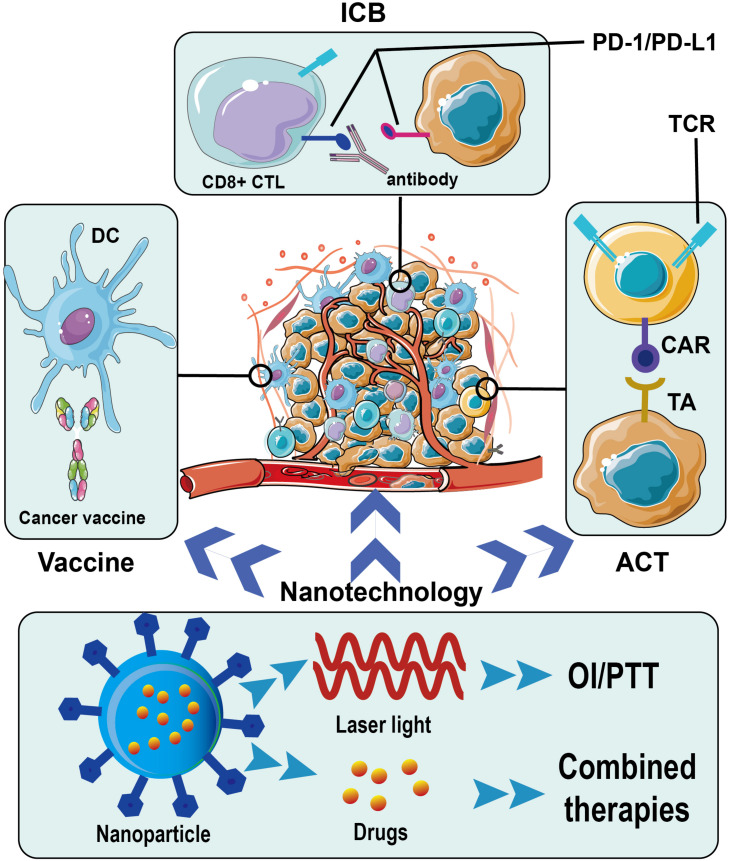
Immunotherapy for breast cancer and application of nanotechnology. ICB, immune checkpoint blockade; PD-1, programmed death protein 1; PD-L1, programmed death protein-ligand 1; DC, dendritic cell; CTL, cytotoxic T lymphocyte; TCR, T cell receptor; CAR, chimeric antigen receptor; TA, tumor antigen; ACT, adoptive cell therapy; OI, optical imaging; PTT, photothermal therapy.

In this review, we summarize the latest progress in immunotherapy for breast cancer and highlight some ongoing clinical trials of various immunotherapeutic approaches on breast cancer. Immune checkpoint blockades (ICBs), adoptive cell therapy (ACT), cancer vaccines, and relevant nanotechnology are mainly discussed (Figure 1).

## Predictive Biomarkers For BC Immunotherapy

Biomarkers have been a hot part of tumor immunotherapy, and the appropriate biomarkers can greatly contribute to the efficiency of tumor immunotherapy. Here, we briefly list a few clinically approved or promising biomarkers under research.

In trials such as Impassion 130 and KEYNOTE-355, PD-L1-positive patients responded significantly better to immunotherapy than others, making it the most widely used biomarker in clinical practice (Schmid et al., 2018, 2019; Cortes et al., 2020). In a phase Ib clinical trial combining durvalumab with trastuzumab in heavily pretreated HER2^+^/PD-L1^–^ metastatic BC, no responses were seen and evidence of cytotoxic T-cell exhaustion was found (Chia et al., 2019). These results indirectly confirmed the role of PD-L1 as a crucial selection biomarker. However, assessment of PD-L1 expression status varied in different trials, and finding the optimal assessment protocol of PD-L1 expression to identify the population that will benefit most from immunotherapy should be a future endeavor.

TMB is another promising predictor of the response to immunotherapy among different cancers (Goodman et al., 2017). As evaluated by Barroso-Sousa et al., TMB varies among different BC subtypes (HR-/HER2^+^> TNBC > HR^+^/HER2^+^> HR^+^/HER2^–^, *P* < 0.05). In a multivariate Cox regression analysis, TMB was the only independent prognostic factor for good metastatic overall survival after adjusting for age and recurrence (Park et al., 2018). Recent research showed that TMB-high tumors were associated with significantly better overall survival (OS) in immune-inflamed TME but considerably worse OS in immunologically cold TME (Thomas et al., 2018).

Interferon γ (IFN-γ)is one of the essential effector molecules in antitumor immunity, and the loss of its receptor was found to completely abrogate the efficaciousness of ICB (Shi et al., 2016). Additionally, the PD-L1 expression and IFN-γ signature exhibited a positive correlation, and PD-L1 and HLA class I expression levels were significantly upregulated in the cases with positive expression status of phosphorylated signal transducer and activator of transcription 1 (one of the IFN-γ signaling pathway molecules) (Nakayama et al., 2019).

Tumor infiltrating lymphocytes (TILs) is also a predictor of immunotherapy effectiveness and has been widely studied (Wein et al., 2017; Wu and Dai, 2017; Tiainen et al., 2019). From a meta-analysis of 3,771 patients, Denkert et al. (2017) found that TILs were able to predict the prognosis and response to chemotherapy among all BC subtypes, especially TNBC and HER2-positive BC (Badr et al., 2019). In recent research, Stromal TILs could identify a subset of stage I TNBC patients with an excellent prognosis without adjuvant chemotherapy. Different types of TILs have reported having different values for predicting prognosis. Researchers found that high levels of TILs expressing programmed death protein 1(PD-1) or FOXP3^+^ TILs (such as Tregs) were correlated to a poor prognosis, yet higher levels of CD8^+^ cytotoxic T lymphocytes predict a good prognosis (Yu et al., 2016; Vihervuori et al., 2019).

FDA approved pembrolizumab for patients with high microsatellite instability/different mismatch repair deficiency advanced/metastatic (MIS/dMMR) solid tumors that have progressed after prior therapy and have no satisfactory alternative treatment options, which made MSI/dMMR a compelling biomarker in solid tumors (Lemery et al., 2017; Yoshino et al., 2020). However, MIS/dMMR was found infrequent in BC, suggesting that it may be inefficient to help identify patients likely to benefit from ICB (Ren et al., 2021; Wu et al., 2021).

Although such biomarkers have been widely assessed and even clinically approved in a variety of solid tumors, no reliable biomarkers for immunotherapy have been established in BC until now. More effort should be put into refining and systematically combining existing biomarkers or finding more efficient biomarkers.

## Immune Checkpoint Blockades

Immune checkpoints are a group of regulators of the adaptive immune system, taking a crucial role in self-tolerance and immune homeostasis. However, they also work as immunosuppressive factors and proved to be associated with chemotherapeutic resistance, thus the research and development of ICBs have greatly promoted the development of immunotherapy. Among ICBs explored in breast cancer, PD-1/PD-L1 blockades catch the most attention.

### PD-1/PD-L1 Blockades

PD-L1 is highly expressed in some BC subtypes, especially in TNBC (with an expression of about 20%), and its expression is correlated to the degree of malignancy (Mittendorf et al., 2014). What’s more, in some cases, PD-L1 expression can be upregulated by conventional therapies including chemotherapy and radiotherapy (Peng et al., 2015; Morisada et al., 2018). Thereby, recently, the studies and clinical trials on PD-1/PD-L1 blockades are almost combined therapies focused on TNBC. Pembrolizumab and nivolumab are the common monoclonal antibodies targeting PD-1 used in clinical trials, while atezolizumab, durvalumab and avelumab are targeting PD-L1.

Conventional anti-tumor therapies were determined with potential immunomodulatory effects and could create a better microenvironment for ICB to obtain better therapeutic effects. For example, in a nivolumab randomized trial (Phase II) in TNBC patients, the results demonstrated that a more favorable tumor microenvironment was achieved by following induction therapy with either chemotherapy or irradiation, increasing the likelihood of response to PD-1 blockade in TNBC, while the majority of responses were observed in the cisplatin (objective response rate (ORR) 23%) and doxorubicin (ORR 35%) cohorts (Voorwerk et al., 2019).

Currently, ICB plus chemotherapy is the most widely explored combination and has achieved striking clinical transformation. As is illustrated in the first and second interim analysis of IMpassion 130, there was a significant benefit in overall survival (OS) in patients with PD-L1 immune cell-positive disease and no obvious difference in OS between the treatment groups in the intention-to-treat population (Schmid et al., 2018, 2019). Besides, a pronounced improvement in progression-free survival was observed in another phase III clinical trial on previously untreated locally recurrent inoperable or metastatic TNBC (KEYNOTE-355) (Cortes et al., 2020), using pembrolizumab plus chemotherapy. Based on these findings, atezolizumab and pembrolizumab were approved by the FDA for PD-L1 positive unresectable, locally advanced or metastatic TNBC in combination with chemotherapy (nab-paclitaxel).

Radiotherapy, one of the most commonly used local treatments for breast cancer, is also being studied coupling with PD-1/PD-L1 blockades. In a phase II clinical trial (NCT02730130), the efficacy of pembrolizumab plus radiotherapy in metastatic TNBC patients was assessed. The ORR for the entire cohort was 17.6% (3 of 17 patients; 95% CI, 4.7–44.2%). Among the nine women assessed by RECIST version 1.1 at week 13, 3 (33%) achieved a CR, with a 100% reduction in tumor volume outside of the irradiated portal, which showed the “abscopal effect” of radiotherapy. Four grade 3 adverse events were reported (Ho et al., 2019). ICBs combined with RT were determined to be safe and demonstrated as a potential approach to patients with poor-prognosis, metastatic TNBC who were unselected for PD-L1 expression.

Ablation treatment for BC mainly includes cryoablation and thermal ablation represented by radiofrequency ablation, which was described to have immunomodulatory effects by recent studies. Immune activation features such as upregulation of inflammatory cytokines, tumor necrosis factor-α, heat shock protein (HSP) as well as downregulation of Tregs were observed after radiofrequency ablation (Schmitt et al., 2007; Fietta et al., 2009; Erinjeri et al., 2013), and were more pronounced after cryoablation (Ahmad et al., 2010). Ablation, as with chemotherapy, could leading to the release of damage-associated molecular pattern molecules like HSP that can sensitize tumors to ICB treatment (Zitvogel et al., 2013; Pfirschke et al., 2016). The ablation therapy in combination with ICB was also a popular modality under preclinical studies and may become a breakthrough in the future.

Despite the unprecedented improvement was achieved in various combinations, more studies and clinical trials are needed to expand indications of PD-1/PD-L1 Blockades in BC. Some other selected clinical trials on anti-PD-1/PD-L1 monotherapy or combination therapy with other agents were summarized in Table 1.

**TABLE 1:** List of some selected trials assessing anti-PD-1/PD-L1 in BCs.

Combined therapy	Anti-PD-1/PD-L1	Another agent	Phase	n	Result	Conclusion	NCT number	References
HER-2-targeted	Pembrolizumab	Trastuzumab	I/II	58	OR (15%, PD-1^+^; 0%, PD-1^–^). fatigue 21%	Safe; with activity and durable clinical benefit in PD-L1^+^/HER2^+^, trastuzumab-resistant, advanced BC patients.	NCT02129556	Loi et al., 2019
PARPi	Pembrolizumab	Nariparib	I/II	55	ORR (21%, total; 47%,brca mutation; 11%,brca wild type), DCR(49%, total; 80%, brca mutation; 33%, brca wild type), median PFS (8.3 m, brca mutation; 2.1 m, X wild type).rca wild typeti	Safe; promising antitumor activity in patients with advanced or metastatic TNBC; higher response rates in BC with tumor BRCA mutations; warranting further investigation.	NCT02657889	Vinayak et al., 2019
/	Pembrolizumab	/	II	170	ORR (5.3%, total; 5.7%, PD-1^+^),DCR (7.6%, total; 9.5%,PD-1^+^) Median PFS 2.0 m; Median OS 9.0 m.	Safe; durable antitumor activity in a subset of patients with previously treated metastatic TNBC.	NCT02447003 (cohortA)	Adams et al., 2019b
Chemotherapy	Atezolizumab	Nab-paclitaxel	Ib	33	ORR 39.4%, DCR 51.5%, Median PFS 5.5 m,OS 14.7 m, DCR 51.5%, Medi	Safe; nab-paclitaxel neither changed biomarkers of the TME (PD-L1, TILs, CD8) nor impaired atezolizumab systemic immune activation.	NCT01633970	Adams et al., 2019a
/	Avelumab	/	Ib	168	ORR (3.0% overall; 5.2%, TNBC). ORR in the overall population (16.7%, PD-1^+^; 1.6%, PD-1^–^), ORR in the TNBC (22.2%, PD-1^+^; 2.6%, PD-1^–^). ≥GRADE 3 AE 13.7%	Safe; clinical activity in a subset of patients with MBC.PD-L1 expression in tumor-associated immune cells associated with a higher probability of clinical response to avelumab in metastatic BC	NCT01772004	Dirix et al., 2018
Chemotherapy	Durvalumab	Anthracycline/taxane	II	117	PCR (53.4%, Durvalumab; 44.2%, placebo), OR = 1.45. Durvalumab effect was seen only in the window cohort (pCR 61.0% vs. 41.4%, OR = 2.22)0.47% thyroid dysfunction.	The combination increases pCR rate particularly in patients treated with durvalumab alone prior to start of chemotherapy increased pCR were observed with higher sTILs. There was a trend for increased PCR rates in PD-L1^+^ tumors.	NCT02685059	Loibl et al., 2019

*BC, breast cancer; TNBC, triple negative breast cancer; RT, radiotherapy; PD-1, programmed death protein 1; PD-L1, programmed death-ligand 1; n, numbers; HER-2, human epidermal growth factor receptor-2; PARPi, poly ADP-ribose polymerase inhibitor; ICB, immune checkpoint blockade; OR, overall response; ORR, objective response rate; DCR, disease control rate; PFS, progression-free survival; AE, adverse event; OS, overall survival; TME, tumor microenvironment; TILs, tumor infiltration lymphocytes.*

### Other Immune Checkpoint Blockades

CTLA-4 is the first immune checkpoint clinically confirmed to be expressed by T cells. It can bind to CD80 and CD86 present on dendritic cells, which restrain the T-cell mediated immune response. There are two main anti-CTLA-4 antibodies: tremelimumab and ipilimumab. In a phase I study of local radiation and tremelimumab in patients with inoperable locally recurrent or metastatic BC, the best response was stable disease, and the median OS was 50.8 months. Moreover, increasing proliferating (Ki67^+^) Treg cells 1-week post-treatment was seen in five patients by peripheral blood mononuclear cell profiles (Jiang et al., 2019). In a pilot study explore cryoablation with ipilimumab in patients with early stage BC, the results suggested the possibility for induced and synergistic anti-tumor immunity with this strategy (McArthur et al., 2016). Anti-CTLA-4 combined with radiotherapy appears to be tolerable, thereby, more research is in need to optimize this combination therapy.

Lymphocyte activation gene-3 (LAG-3) is a newly discovered inhibitive receptor that is highly expressed in Tregs and disabled T cells. It can provide inhibitive signals to activated effector T cells and enhance the blocking activity of Treg. The anti-LAG-3 antibody can not only activate effector T cells but also inhibit the activity of Tregs, while anti-PD-1 and anti-CTLA-4 antibodies can only activate effector T cells. The result obtained from a completed phase I clinical trial on 30 metastatic BC patients treated with chemo-immunotherapy using IMP321 (a recombinant soluble LAG-3Ig fusion protein) plus paclitaxel indicated a dramatic improvement in ORR (Brignone et al., 2010). Safety and efficacy of the immunotherapy IMP321 in combination (adjunctive) with paclitaxel chemotherapy in patients with hormone receptor-positive metastatic BC are under investigation in an ongoing phase 2 clinical trial (NCT02614833).

## Cancer Vaccines

The cancer vaccine is a kind of vaccine that mobilizes the immune system to produce cytotoxic T-lymphocytes with anti-tumor effect and long-term memory CD8^+^ T cells, while the functional activity of producing a CD8^+^ /CD4^+^ T cell response is restricted by human leukocyte antigen (HLA) (Nishimura et al., 2015). The vaccines present either a single tumor-associated/specific antigen to the immune system (monovalent vaccine), or provide immunity against multiple immunogens (Behravan et al., 2019). In addition to peptides, antigen-presenting cells such as dendritic cells or modified tumor cells can be used as preparation material for cancer vaccines (Arab et al., 2019).

Currently, the exploration of cancer vaccine on BC is mainly focused on the protein/polypeptide vaccine, especially HER2, of which the anti-E75 (HER2/neu 369-377) vaccine is the most popular. In a systematic review and meta-analysis of 16 trials, the cancer recurrence was decreased, while the OS and disease-free survival of patients were significantly improved, in the vaccinated subjects vs. the control cohort (Chamani et al., 2018). The security of HER2 vaccines was confirmed in another meta-analysis summarizing the toxicity profiles of HER2 vaccines (Costa et al., 2019). Such results promoted relevant clinical researches.

Initial results for many vaccines varied among different research, while some patients benefited from them but the others not, which showed the possibility of vaccines as a personalized treatment for BC patients. In a phase I trial on resectable HER2^+^ breast cancer (Knutson et al., 2019), kinds of research developed a degenerate HER2 epitope-based vaccine consisting of four HLA class II-restricted epitopes mixed with granulocyte-macrophage colony stimulating factor. Only two experienced disease recurrence. The percent of patients that responded with augmented T cell immunity was high for each peptide ranging from 68 to 88%. The vaccine also augmented HER2-specific antibodies with sustained immune activation during the next 2 years after vaccination. However, the result obtained from another phase III trial on Nelipepimut-S Vaccine (a cancer vaccine comprised of HLA A2/A3 restricted HER2/neu) indicated acceptable tolerance but no significant clinical benefit in preventing BC recurrence (Mittendorf et al., 2019).

Given the low immunogenicity of various cancer vaccines and heterogeneity of cancer, various approaches have been explored to raise the efficacy of cancer vaccines. A lysosome-associated membrane protein 1 (LAMP) domain had been utilized to enhance vaccine efficacy against HER2, as an alternative antigen trafficking, in both *in vitro* and *in vivo* studies. As a result, HER2^–^ LAMP vaccine produced tumor regression in 30% of vaccinated mice with an endogenous model of metastatic HER2^+^ BC, by enhanced major histocompatibility complex (MHC) class I and II presentation that elevated levels of antigen-specific polyfunctional CD8^+^ T cells (Chen et al., 2020). Additionally, another oncolytic vesicular stomatitis virus-based whole-cell cancer vaccine also improves TNBC outcome in a mouse model by enhancing natural killer and CD8^+^ T cell functionality (Niavarani et al., 2020).

## Adoptive Cell Therapy

ACT is the transfer of sensitized lymphocytes (such as TILs, CD8^+^ cells, CD4^+^ helper cells, and NKs, etc.) which have been manipulated and amplified *in vitro* to cancer patients to obtain anti-tumor immunity. It has become highly promising immunotherapy to induce the anti-tumor activity of the host immune system, especially in patients with low immunity.

Cytokine-induced killers (CIK) cells are mainly CD3^+^ CD56^+^ cytotoxic lymphocytes with anti-tumor activation and no MHC restriction, thereby they can kill tumor cells directly and promote T cell proliferation. A retrospective study of immunotherapy with autologous CIK cells in 294 BC patients indicated that longer disease-free survival and OS intervals were associated with an increased number of CIK treatment cycles, and chemotherapy in combination with adjuvant CIK was promising to extend the survival time of TNBC patients, especially those at early stages (Zhang et al., 2019). However, the benefit was only seen in the patients with PD-L1 expression (Zhou Z. Q. et al., 2019), which indicated the relationship between PD-L1 expression and CIK therapy. PD-L1 expression could serve as an indicator of adjuvant CIK therapy for breast cancer after operations. The combination of CIK and anti-PD-1 therapy may be a new research direction.

Chimeric antigen receptor T cell (CAR-T) therapy is an innovative form of immunotherapy in which autogenous T cells are genetically modified to express chimeric receptors encoding antigen-specific fragments and various co-stimulating molecules. Unlike physiological T cells with common T cell receptors, these modified CAR-T cells are transported to and recognize cancer cells in ways unrelated to HLA. Presently, CAR-T therapy has performed well in the treatment of hematologic malignancies, but challenges remain in solid tumors such as breast cancer, with scarce targeted antigens and limited duration.

Many targets are under study for CAR-T cell therapy for BCs. For example, anti-HER2 CAR-T cells (NCT02547961), anti-NKG2DL CAR-T cells (NCT04107142), and anti-cMET CAR-T cells (NCT03060356) have been assessed in clinical trials. TEM8 is a tumor endothelial marker initially found in colon cancer and also highly expressed in TNBC. It was shown that anti-TEM8 CAR-T cells could induce regression of metastatic TNBC by killing TEM8 ^+^ TNBC tumor cells and targeting the tumor endothelium to block tumor neovascularization (Byrd et al., 2018). In a xenograft model (Zhou R. et al., 2019), CAR T cells modified by TAB004, a monoclonal antibody that is highly specific for the tumor-associated mucin1 glycoprotein (MUC1), was capable to increase the production of Granzyme B, IFN-γ, and other Th1 type cytokines, chemokines and significantly reduced the progression of tumor-associated MUC1 positive TNBC. Additionally, Such CAR-T cells did little damage to normal breast epithelial cells, because TAB004 could differentiate MUC1 between cancerous cells and normal cells. In some BC subtypes, the highly expressed targets like epidermal growth factor receptor were promising used for CAR-T cell therapies to inhibit the advance of cell-line and patient-derived xenograft TNBC (Liu et al., 2019).

Emerging strategies have been developed to perfected CAR-T therapy. A microphysiologic 3D tumor model has been developed as a standardized, scalable test system to improve and evaluate the antitumor activity of CAR-T cells for their preclinical safety and efficacy (Wallstabe et al., 2019). Meanwhile, a new generation of CAR constructs capable of inducing cytokine signaling after antigen stimulation has been designed, which showed superior *in vivo* persistence and antitumor effects in models of solid tumors, compared with traditional CAR-T cells only expressing a CD28 co-stimulatory domain (Kagoya et al., 2018). Considering that the interactions between PD-1 and activated T cells as well as its ligands and a target tumor may suppress the function of CAR-T cells to kill solid tumor cells, combining CAR-T therapy with PD-1/PD-L1 blockades was excepted to be synergistic. And in a recent study, CAR-T cells with PD-1 disruption performed better control of tumor and recrudescence prevention *in vivo*, compared with those without anti-PD-1 (Hu et al., 2019). Based on such preclinical experimental results, more clinical trials are needed to further verify its effectiveness and safety.

## Nanotechnology for Immunotherapy

With the development of new nanoparticles for cancer diagnosis and treatment, unprecedented improvement have been achieved in the field of nano-immunotherapy. Unique properties of nanoparticles allows them to deliver various compounds such as different imaging and therapeutic drugs (Thakor and Gambhir, 2013). Nanoparticles was able to efficiently concentrate in tumor by passive targeting (based on the enhanced permeability and retention effect) (Matsumura and Maeda, 1986) and/or active targeting (Mi et al., 2020). Moreover, some nanoparticles were also used for photothermal or photodynamic therapy.

Recent advances in nanotechnology and biomedical engineering enable nano-drug delivery systems targeting the TME being anticipated to revolutionize cancer treatment. A biocompatible cubic-phase (α-phase) erbium-based rare-earth nanoparticles (ErNPs), exhibiting bright downconversion luminescence at 1,600 nm, was developed for dynamic imaging of cancer immunotherapy in mice (Zhong et al., 2019). Combining 1,600 nm-emitting ErNPs conjugated with anti-PD-L1 monoclonal antibody and lead sulfide quantum dots conjugated with anti-CD8α monoclonal antibody, the two-plex molecular imaging revealed heterogeneous bio-distributions of PD-L1 and CD8^+^ CTLs. Tumor/normal tissue ratios of PD-L1 were high in CT-26 colon tumors with positive therapeutic responses to anti-PD-L1 therapy, but lower in non-responding 4T1 tumors (Zhong et al., 2019). Thus, combining *in vivo* tumor PD-L1 expression level and immune cell status to predict the immunotherapeutic reaction could be promising to distinguish patients who may benefit from immunotherapy.

Some nanoparticles with photothermal and/or photothermal effect in combination with immunotherapy have shown better efficacy in BC (Tian et al., 2019; Zhuang et al., 2020). Our group has designed a JQ1 (bromodomain and extra-terminal blockade)-Loaded Polydopamine Nanoplatform, capable of combining immunotherapy and photothermal therapy, which significantly increased the activation of CTLs and strongly induced immune-memory effect to counter cancer relapse (Tian et al., 2019). Similarly, an intelligent biomimetic nanoplatform loaded with copper sulfide, immune adjuvant resiquimod and a homogenous cancer cell membrane, could synergistically exert photothermal ablation and immune remodeling to prevent TNBC recurrence and metastasis (Cheng et al., 2020).

In addition to specifically targeting tumors, neoantigen-based nanoplatforms could effectively accumulate in lymph nodes to improve antigen presentation efficiency, and co-deliver immunomodulatory agents to promote the generation and effects of anti-tumor immunity. For example, using a nanoplatform to deliver both neoantigen and interferon gene stimulation pathway activator, researchers significantly suppressed tumor growth in 4T1 breast cancer mice and produced robust anti-tumor effects in combination with PD-L1 inhibitors (Zhou et al., 2020).

How to convert the immune-depleted TME into an immunogenic TME for corresponding immunotherapy has been a popular topic in breast cancer immunotherapy. Researchers effectively improved the TME and eliminated lung metastases in combination with anti-PD-1 in 4T1 mice models, using self-assembled nanoliposomes to deliver adriamycin and indoximod (a phospholipid-conjugated prodrug inhibiting the indoleamine 2, 3-dioxygenase pathway) (Lu et al., 2018). Chinese researchers delivered the chemotherapeutic agent SN38 (7-ethyl-10-hydroxycamptothecin) and the STING agonist into tumors using nanoparticles, and observed conversion of the immunosuppressive TME to immunogenic TME. Additionally, remarkable therapeutic benefit was achieved when combined with anti–PD-1 therapy (Liang et al., 2020).

Despite the achievement of nanotechnology for immunotherapy, more attention should be paid to the development of nanoparticles with good biocompatibility and theranostic ability.

## Conclusion

In conclusion, well-established biomarkers and reasonable patient stratification are critical to the effectiveness of immunotherapy, and combination therapy will be the way forward for BC immunotherapy. How to determine the best strategy will be a long-term challenge. Improved understanding of the interplay between tumor and microenvironment is expected to provide new directions for therapeutic strategies. The development of nanomedicine showed great potential in the era of immunotherapy of BC, such as combining multimodality therapies, stratification of patients and outcome prediction. Nano-immunotherapy may become one of the hot spots for future breast cancer research.

## Author Contributions

SW and YJ presented the initial idea for the research and provided financial support. YL, WM, and DH wrote most of the article. SW, YJ, JL, and SW reviewed the article and gave valuable suggestions for revising the article. All authors contributed to the article and agreed with the submission version.

## Conflict of Interest

The authors declare that the research was conducted in the absence of any commercial or financial relationships that could be construed as a potential conflict of interest.
